# Macronutrient balance dictates lifespan and reproduction in a beetle, *Tenebrio molitor*

**DOI:** 10.1242/jeb.250281

**Published:** 2025-06-20

**Authors:** Myung Suk Rho, Kwang Pum Lee

**Affiliations:** Department of Agricultural Biotechnology and Research Institute of Agriculture and Life Sciences, Seoul National University, 1 Gwanak-ro, Gwanak-gu, Seoul 08826, Republic of Korea

**Keywords:** Ageing, Carbohydrate, Coleoptera, Fecundity, Geometric framework, Life-history trade-off, Mealworm beetle, Protein

## Abstract

Macronutrients profoundly affect both lifespan and reproduction and also modulate the fundamental trade-off between these two components of fitness in many insects. Beetles represent the largest group of insects, but nutritional interventions in lifespan and reproduction have never been thoroughly explored in this taxon. Here, we used nutritional landscape methodology to determine the effects of protein and carbohydrate intake on lifespan and reproduction in the mealworm beetle, *Tenebrio molitor* (Coleoptera: Tenebrionidae). The time of death and last reproduction, the number of eggs laid and protein–carbohydrate intake were recorded from beetles maintained on one of 35 chemically defined foods varying in protein-to-carbohydrate ratio (P:C=0:1, 1:5, 1:2, 1:1, 2:1, 5:1 or 1:0) and in protein plus carbohydrate concentration (P+C=25.2%, 33.6%, 42%, 50.4% or 58.8%). Lifespan and reproductive traits increased with higher caloric intake, but their respective trait maxima occurred at different P:C ratios. Female reproductive traits peaked at higher P:C ratios (reproductive lifespan 1:1.06; lifetime egg production 1.31:1; egg production rate 1.75:1) than those maximizing lifespan (male lifespan 1:1.38; female lifespan 1:1.36). This divergence indicates a nutrient-mediated trade-off between lifespan and reproduction in this species. Despite this, the nutritional conflict in *T. molitor* appeared to be less pronounced than what has been observed in other species commonly used in insect ageing research. When given a food choice, *T. molitor* beetles selected a P:C ratio close to 1:1, which simultaneously supported extended lifespan and high reproductive output.

## Teaser

**Summary:** Macronutrient balance is a critical determinant of longevity and reproductive output in the mealworm beetle, *Tenebrio molitor*.

## INTRODUCTION

Understanding how diet impacts lifespan and the process of ageing has long been a central theme in life-history and ageing research (Fontana et al., 2010; Fontana and Partridge, 2015). The most well-known example of dietary intervention in ageing is the extension of lifespan through moderate restriction of food intake – a phenomenon widely known as dietary restriction (DR) (Masoro, 2005; Mair and Dillin, 2008; Speakman and Mitchell, 2011). Lifespan extension by DR has been documented across a wide range of organisms spanning from yeasts to primates and is often reported to come at the expense of reduced reproduction (Chapman and Partridge, 1996; Shanley and Kirkwood, 2000; Partridge et al., 2005). It has been traditionally held that this negative association between survival and reproduction arises because these two components of fitness compete for a finite pool of resources, with increased investment of resources in one trait leading to reduced investment in the other (Kirkwood, 1977; Shanley and Kirkwood, 2000). This resource allocation hypothesis posits that, when food availability declines, organisms redirect their limited resources away from reproduction toward somatic maintenance and repair, thereby promoting lifespan in exchange for reproduction. This mode of resource allocation is thought to be an adaptive strategy for surviving periods of food scarcity. When food availability improves, the reverse pattern is expected – greater investment in reproduction accompanied by a reduction in lifespan. In recent years, however, this conventional explanation for the lifespan-promoting effect of DR has come under scrutiny, as a growing number of studies have highlighted the crucial role of specific nutrients in shaping the relationship between lifespan and reproduction (Nakagawa et al., 2012; Moatt et al., 2020; Piper et al., 2023).

Nutrition is highly complex in nature, comprising multiple interacting components that exert both linear and non-linear effects on organismal performance, health and evolutionary fitness (reviewed by Simpson and Raubenheimer, 2012). The Geometric Framework (GF) is a powerful state–space modelling framework that offers a unified platform for analysing highly complex multidimensional nutritional datasets (Simpson and Raubenheimer, 2012). The use of the GF has allowed us to visualize the phenotypic consequences of ingesting multiple nutritional components (e.g. protein and carbohydrate) as a nutritional performance landscape (nutritional landscape, hereafter). When the nutritional landscape for lifespan was mapped in *Drosophila* flies (Lee et al., 2008; Jensen et al., 2015; Semaniuk et al., 2018; Jang and Lee, 2018; Carey et al., 2022) and many other insects (Maklakov et al., 2008; Fanson et al., 2009; Archer et al., 2015; Malod et al., 2017; Rapkin et al., 2017; Ng et al., 2018, 2019; Hawkes et al., 2022), it became apparent that lifespan patterns were predominately explained by the balance of macronutrients (e.g. protein and carbohydrate), rather than by total caloric intake. This finding challenges the long-standing view that reduced caloric intake is the primary driver of the lifespan-extending effects of DR (Lee et al., 2008; Piper et al., 2011; Nakagawa et al., 2012; Le Couteur et al., 2016; Simpson et al., 2017; Moatt et al., 2019).

Application of the GF has also provided important insights into the role of macronutrient intake in determining the negative association between lifespan and reproduction. It has been demonstrated in many organisms that lifespan is maximized on a diet low in protein and high in carbohydrate whereas female fecundity is maximized on a diet high in protein and low in carbohydrate (reviewed by Simpson and Raubenheimer, 2012; Le Couteur et al., 2016). The fact that lifespan and reproduction are maximized in different regions in the nutrient space suggests that these two components of fitness require different macronutrient intake for expressing their trait maxima and therefore cannot be simultaneously maximized at the same macronutrient intake. These findings have laid the foundations for a new hypothesis, which posits that the fundamental life-history trade-off between lifespan and reproduction is mediated by the composition of macronutrients consumed, rather than by a competitive resource allocation of finite resources (Simpson and Raubenheimer, 2012). In recent years, growing efforts have been made to develop formal methods to quantify the occurrence and strength of this nutrient-mediated or nutrient space-based trade-off between lifespan and reproduction (Rapkin et al., 2018; Morimoto and Lihoreau, 2019; del Castillo et al., 2022; Morimoto et al., 2023).

To date, a growing body of research has used nutritional landscapes to illuminate the effects of multiple macronutrients (protein, carbohydrate and lipid) on lifespan, reproduction and their trade-off in insects. However, most of these investigations have concentrated on two insect groups: flies from the order Diptera (Lee et al., 2008; Fanson et al., 2009; Jensen et al., 2015; Malod et al., 2017; Semaniuk et al., 2018; Jang and Lee, 2018; Carey et al., 2022) and crickets from the order Orthoptera (Maklakov et al., 2008; Harrison et al., 2014; Archer et al., 2015; Rapkin et al., 2017; Ng et al., 2018, 2019; Hawkes et al., 2022). These two groups of insects have repeatedly demonstrated the case of strong nutrient-mediated trade-offs between lifespan and reproduction. However, there remain concerns over whether results derived almost exclusively from these two insect groups might have skewed our understanding of the relationships among nutrition, lifespan and reproduction. Hence, to understand whether such strong nutrient-mediated trade-offs are universal across insect taxa, more GF studies are needed on insect species representing a wider range of taxonomic groups. Beetles, belonging to the order Coleoptera, constitute the largest and most taxonomically diverse group of insects. Yet, no studies have comprehensively mapped the nutritional landscape required to assess the occurrence and strength of nutrient-mediated trade-offs between lifespan and reproduction in beetles.

The mealworm beetle, *Tenebrio molitor* Linnaeus (Coleoptera: Tenebrionidae), has been widely used as a model organism in many areas of fundamental research, including biochemistry, physiology, immunology and behavioural ecology (Rutowski, 1982; Vigneron et al., 2019; Kojour et al., 2022), and has also emerged as one of the most economically important food and feed insects (Ghaly and Alkoaik, 2009; Grau et al., 2017; Ribeiro et al., 2018). Until recently, lifespan in *T. molitor* had been studied primarily in the context of its relationship with immunity and sexual selection (Armitage et al., 2003; Krams et al., 2016; McConnell and Judge, 2018), but it had rarely been the subject of insect ageing research. In particular, it remains largely unexplored how nutrition affects lifespan and its associated life-history traits, such as reproduction, in this species. Previously, Rho and Lee (2016) examined the lifespan and egg production of *T. molitor* beetles confined to one of three isocaloric diets differing in protein-to-carbohydrate ratio (P:C=1:5, 1:1, 5:1) and found that these two traits were simultaneously maximized on a diet with a P:C ratio of 1:1. However, this simplistic approach, which tested only three P:C ratios, could not provide sufficient resolution to detect any divergence that may actually exist between the nutritional optima for lifespan and reproduction in this species.

In this study, we constructed nutritional landscapes to illustrate the linear and non-linear effects of protein and carbohydrate intake on lifespan and reproduction in *T. molitor*. The amounts of protein and carbohydrate consumed, lifespan and various parameters of female reproductive performance were recorded from individual beetles that were fed one of 35 experimental diets that varied systematically in protein and carbohydrate content. The topographical patterns of the nutritional landscapes mapped for lifespan and female reproductive traits were then compared to evaluate the occurrence and strength of nutrient-mediated trade-offs between lifespan and reproduction, using recently developed analytical tools (Morimoto and Lihoreau, 2019; del Castillo et al., 2022; Morimoto et al., 2023). Having established the nutritional landscapes for major fitness related traits, we then performed a food choice assay to test whether female *T. molitor* beetles would select an optimal balance of protein and carbohydrate that maximizes their fitness or lifetime reproductive success (Lee et al., 2008; Jensen et al., 2012).

## MATERIALS AND METHODS

### Synthetic diets

Based on the protocol of Simpson and Abisgold (1985), we prepared a total of 35 chemically defined diets that contained one of seven P:C ratios (0:1, 1:5, 1:2, 1:1, 2:1, 5:1 or 1:0), with five protein plus carbohydrate (P+C) concentrations (25.2%, 33.6%, 42%, 50.4% or 58.8% by dry mass) for each ratio. The protein component of these diets comprised a 3:1:1 mixture of casein, peptone and albumen whereas sucrose was the sole source of digestible carbohydrate. Protein and carbohydrate were diluted with indigestible cellulose. Each diet contained fixed concentrations of 2.5% Wesson salt, 0.5% cholesterol, 0.5% linoleic acid, 0.3% ascorbic acid and 0.2% vitamin mixture (Rho and Lee, 2023). The diets were provided to the insects in the form of a dry powder.

### Protocol

To obtain experimental insects, approximately 500 mixed-sex adult *T. molitor* beetles were collected from a large outbred stock culture maintained at Seoul National University, Seoul, Republic of Korea, and placed in oviposition trays (20 cm×17 cm×11 cm) filled with wheat bran. Eggs laid on these trays over 24 h were harvested and incubated until hatching. Newly hatched larvae were transferred in groups of 300–400 individuals to plastic containers (40 cm×17 cm×11 cm) and reared until pupation on wheat bran at 25°C under a 12 h:12 h light:dark photoperiod. Throughout the larval rearing period, fresh cabbage leaves were provided twice per week. Pupae were collected from the containers, sexed and allowed to complete pupal development at 25°C.

On the day of adult eclosion (day 0), a total of 1800 newly eclosed beetles (900 males and 900 females) were weighed to the nearest 0.1 mg (initial fresh mass) and randomly assigned to 35 no-choice treatments and one choice treatment, with 22–28 replicates per treatment per sex. Beetles assigned to the no-choice treatments received one of 35 diets varying in P:C ratio and P+C concentration, whereas those in the choice treatment were offered a pair of nutritionally complementary diets, one with a protein-biased P:C ratio of 5:1 (P:C=35%:7%) and the other with a carbohydrate-biased P:C ratio of 1:5 (P:C=7%:35%).

Experimental insects were housed individually in their own feeding arenas (5 cm Petri dish) and supplied with either one (no-choice) or two (choice) food dishes (the upturned lid of 1.5 ml Eppendorf tube; 9 mm diameter, 5 mm depth) and water (in a 1.5 ml Eppendorf tube capped with a cotton plug) throughout their lifespan. Prior to being presented to the insects, food dishes were filled with granular diets, dried in an oven set at 40°C for 48 h and weighed. Food dishes were removed and replaced with fresh, pre-weighed ones every 2 days. To ensure accurate measurement of food intake, any spilled food was collected and returned to the dish before removal. Removed food dishes were dried at 40°C for 48 h to eliminate moisture prior to weighing. The amount of food consumed by each insect over every 2 day period was calculated as the difference in dry mass between the initially presented and the collected food dishes. Protein and carbohydrate intake were computed as the product of food intake and the known concentration of each macronutrient in the diet. Most beetles started to eat the synthetic diet on day 4 of adult life. Food intake was measured until day 22. Beetles that did not consume food or died before day 22 were excluded from the data analysis.

When beetles reached sexual maturity on day 8, male and female beetles from the same diet treatment were randomly paired in mating arenas (5 cm Petri dish) and allowed to mate for 24 h (Rho and Lee, 2016). Mating was considered successful if males mounted and remained attached to females by their genitalia for 1–2 min (Font and Desfilis, 2003). We confirmed that all pairs successfully completed copulation within 30 min. After this 24 h mating period, each insect was separated from its partner, returned to its feeding arena and allowed to feed for another 6 days. This cycle was repeated every week until all beetles had died. Throughout the experimental period, beetles copulated with the same partners. In pairs where one partner died earlier, the surviving partner was paired with a new mate that had been maintained on the same diet treatment.

The death of each insect was checked daily, and the number of eggs produced by each female beetle was counted every 2 days. The day on which the last egg was laid was also recorded. Organismal lifespan (hereafter referred to as simply ‘lifespan’) was defined as the number of days between adult eclosion (day 0) and death. For each female beetle, we also measured the length or duration of the reproductive period (i.e. reproductive lifespan), which was calculated as the number of days between adult eclosion and the last day of egg production. Lifetime egg production, which represents lifetime reproductive success, was determined as the total number of eggs produced by each female over its lifespan. Egg production rate or daily reproductive effort was computed as the total number of eggs laid divided by the number of days between the onset of sexual maturity (day 8) and the last day of egg production.

### Statistical analysis

Visualization of nutritional landscapes and estimation of their topographical parameters were performed using R v.3.5.1 (http://www.R-project.org/). All other statistical analyses were conducted using SAS v.9.12 (SAS Institute, Cary, NC, USA).

Non-parametric thin-plate splines were used to construct nutritional landscapes illustrating how each response variable was expressed across a range of protein and carbohydrate intake and were plotted using the *Tps* function in the ‘Fields’ package (Nychka et al., 2017) in R. When mapping each landscape, the smoothing parameters that minimized the generalized cross-validation score (GCV) was selected. For each nutritional landscape, we estimated the location of the trait maximum (or peak) and its 95% confidence regions (CRs) using the *OptRegionTps* function in ‘OptimaRegion’ package in R (del Castillo et al., 2022).

While a non-parametric technique was used to visualize nutritional landscapes, parametric multivariate polynomial regressions were employed to determine the linear and non-linear (quadratic and correlational) effects of protein and carbohydrate intake (mg day^−1^) on the measured response variables (Lande and Arnold, 1983). For each response variable, we first fitted a model including only the linear terms, from which we estimated the linear gradients for protein and carbohydrate. We then added the non-linear (quadratic and correlational) terms to the initial model and re-ran the analysis. Non-linear gradients were estimated from this second model (Jensen et al., 2015). To test whether the linear and non-linear effects of protein and carbohydrate intake differed significantly across the different response variables, we used a sequential model-building approach, performing a series of pairwise partial *F*-tests comparing the models with and without the interaction terms between the response variable and the linear and non-linear terms for protein and carbohydrate intake (see South et al., 2011). Before conducting these pairwise comparisons, each response variable was standardized to account for scaling or unit differences.

The occurrence and strength of nutrient-mediated trade-offs between the measured response variables were analysed by assessing the divergence between their nutritional optima (or peaks) in the nutrient space. The degree of divergence was quantified by calculating the Euclidean distance (*d*) between the peaks for these response variables and the angle (θ) between their position vectors. Greater distances and angles between the peaks were interpreted as indicative of stronger trade-offs between the response variables. The Euclidean distance (*d*) between the two peaks and its 95% confidence interval (CI) were estimated using the *CRcompare* function from the ‘OptimaRegion’ package in R (see Rapkin et al., 2018; Carey et al., 2022). The angle separating the position vectors and its 95% CI were computed using the vector of position approach and nutrigonometry (Morimoto and Lihoreau, 2019; Morimoto et al., 2023). When estimating the peak regions of the nutritional landscapes, the general linear model was applied, as it provided the most accurate predictions of these regions (Morimoto et al., 2023). A significant divergence between the position vectors was inferred if the 95% CI of the estimated angle did not include 0 deg.

To assess whether male and female beetles selected protein and carbohydrate differently when given a food choice, we performed both multivariate and univariate analysis of variance (MANOVA and ANOVA) with sex as the main factor. Pillai's trace statistics were used in the multivariate test.

## RESULTS

### Lifespan

**Fig. 1.**
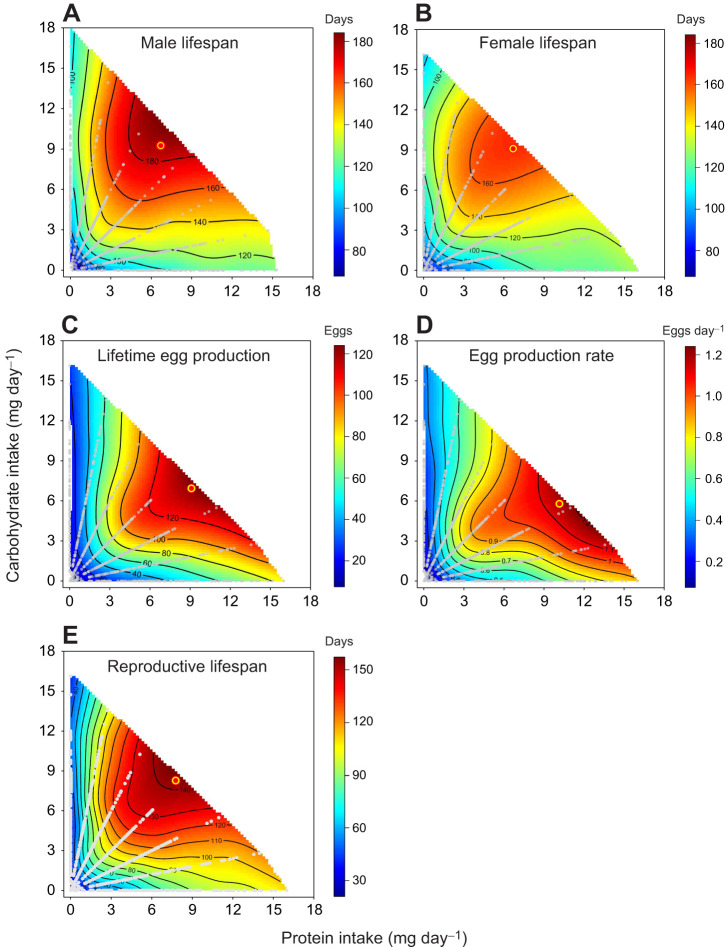
**Nutritional landscapes for lifespan and reproduction.** Effects of protein and carbohydrate intake on (A) male lifespan, (B) female lifespan, (C) female lifetime egg production, (D) female egg production rate and (E) female reproductive lifespan in *Tenebrio molitor* beetles confined to one of 35 chemically defined diets varying in protein and carbohydrate content. In each panel, the bull's-eye marks the position of the peak in the nutrient landscape mapped for the corresponding response variable. Grey dots represent the amount of protein–carbohydrate eaten by individual beetles per day.

Males lived ca.11.3 days longer than females across the 35 diet treatments (mean±s.e.m.: males 111.8±4.44 days, females 100.5±4.40 days; *t*=4.15, d.f.=1653, *P*<0.001). The lifespan of both male and female beetles exhibited significant positive linear terms for protein and carbohydrate intake (Table 1), indicating a trend of increasing lifespan in response to increasing protein and carbohydrate intake. More importantly, we detected significant negative quadratic terms for protein and carbohydrate intake on male and female lifespan (Table 1), suggesting the presence of a peak for male and female lifespan in the nutritional landscape (Fig. 1A,B). Lifespan peaked at a P:C ratio of 1:1.38 (protein 6.70 mg day^−1^, carbohydrate 9.26 mg day^−1^) in males and 1:1.36 (protein 6.69 mg day^−1^, carbohydrate 9.10 mg day^−1^) in females, both at high P+C intake. In both sexes, lifespan decreased progressively as the P:C ratio either increased or decreased from this optimal P:C ratio and also as the P+C intake decreased.

**Table 1.** Linear and non-linear effects of protein (P) and carbohydrate (C) intake on male and female lifespan, female lifetime egg production, female egg production rate and female reproductive lifespan in *Tenebrio molitor* beetles

		Linear effects		Non-linear effects		
		P	C	P^2^	C^2^	P×C
Male lifespan	Gradient±s.e.	4.83±6.78e−01	5.62±6.95e−01	−1.02±1.69e−01	−8.93e−01±1.54e−01	2.92e−01±3.12e−01
	*T*	7.13	8.09	−6.03	−5.79	0.94
	d.f.	850	850	847	847	847
	*P*	<0.001	<0.001	<0.001	<0.001	0.350
Female lifespan	Gradient±s.e.	4.99±5.84e−01	7.38±6.34e−01	−7.63e−01±1.37e−01	−1.16±1.60e−01	−9.75e−02±2.98e−01
	*T*	8.54	11.63	−5.56	−7.27	−0.33
	d.f.	799	799	796	796	796
	*P*	<0.001	<0.001	<0.001	<0.001	0.744
Lifetime egg production	Gradient±s.e.	7.43±6.42e−01	4.02±6.97e−01	−5.58e−01±1.48e−01	−8.18e−01±1.71e−01	1.73±3.20e−01
	*t*	11.56	5.78	−3.78	−4.78	5.41
	d.f.	799	799	796	796	796
	*P*	<0.001	<0.001	<0.001	<0.001	<0.001
Egg production rate	Gradient±s.e.	5.88e−02±6.13e−03	2.37e−02±6.65e−03	−5.71e−03±1.48e−03	−6.58e−03±1.71e−03	7.15e−03±3.20e−03
	*t*	9.59	3.57	−3.87	−3.85	2.23
	d.f.	799	799	796	796	796
	*P*	<0.001	<0.001	<0.001	<0.001	0.026
Reproductive lifespan	Gradient±s.e.	6.69±5.88e−01	4.30E±6.39e−01	−8.49e−01±1.37e−01	−6.91e−01±1.59e−01	9.10e−01±2.96e−01
	*t*	11.37	6.73	−6.22	−4.35	3.07
	d.f.	799	799	796	796	796
	*P*	<0.001	<0.001	<0.001	<0.001	0.002

**Fig. 2.**
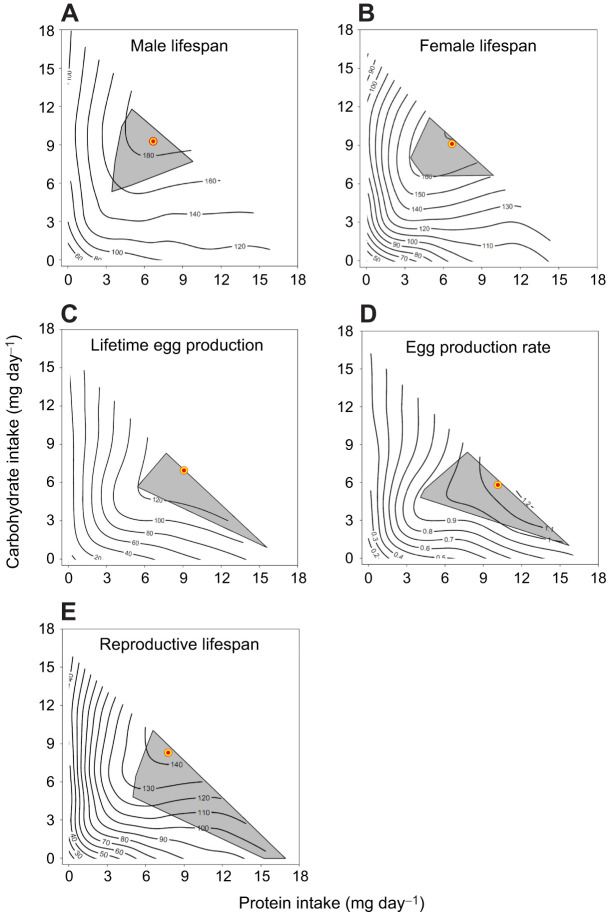
**Confidence regions of nutritional optima for lifespan and reproduction.** The position of the peak (bull's-eye) and its 95% confidence region (grey area) in the contour plots fitted for (A) male lifespan, (B) female lifespan, (C) female lifetime egg production, (D) female egg production rate and (E) female reproductive lifespan in *T. molitor* beetles confined to one of 35 chemically defined diets varying in protein and carbohydrate content.

The shapes of the nutritional landscapes mapped for male and female lifespan (Fig. 1A,B) were qualitatively similar, as evidenced by the lack of significant differences in the effects of protein and carbohydrate intake on lifespan between the sexes (Table 2). The distance (*d*) between the peaks for male and female lifespan was 1.25 mg day^−1^ and the angle (θ) between their position vectors was 3.9 deg (Table 2). As the 95% CI of the angle included 0 deg, the directions of the two position vectors were not significantly different. Furthermore, we found that the 95% CR of the peaks for male and female lifespan overlapped in the central region of the nutrient space (Fig. 2A,B).

**Table 2.** Pairwise comparisons of nutritional landscapes mapped for male and female lifespan in *T. molitor* beetles

	*F*				*d* (mg day^−1^)		θ (deg)	
	Linear	Quadratic	Correlational	Overall	Estimate	95% CI	Estimate	95% CI
Male lifespan versus female lifespan	2.10^n.s.^	1.86^n.s.^	0.79^n.s.^	1.10^n.s.^	1.25	0.91, 1.53	3.90	−12.3, 20.11

*F*-ratios from partial *F*-tests comparing the linear, quadratic and correlational effects of protein and carbohydrate intake on male and female lifespan are presented, along with the Euclidean distance (*d*) between the nutritional optima for male and female lifespan and the angle (θ) between their position vectors. 95% CI, 95% confidence interval (lower, upper); n.s., not significant.

### Egg production

Lifetime egg production in females increased linearly with increasing protein and carbohydrate intake, as indicated by significant positive linear terms for both macronutrients (Table 1). Significant negative quadratic terms for protein and carbohydrate intake on lifetime egg production (Table 1) suggest the presence of a peak for this measure of reproductive success in the nutritional landscape (Fig. 1C). Lifetime egg production was maximized at a P:C ratio of 1.31:1 (protein 9.10 mg day^−1^, carbohydrate 6.94 mg day^−1^) and at high P+C intake. As with lifespan, lifetime egg production decreased as the P:C ratio either increased or decreased from the optimal P:C ratio and as the P+C intake decreased.

Egg production rate also exhibited significant positive linear and negative quadratic terms for protein and carbohydrate intake (Table 1), suggesting the presence of a peak for this response variable in the nutritional landscape (Fig. 1D). The P:C ratio that maximized egg production rate was 1.75:1 (protein 10.16 mg day^−1^, carbohydrate 5.79 mg day^−1^), which was more protein biased than that which maximized lifetime egg production (see above). Despite this difference in the optimal P:C ratio, the distance (*d*) between the peaks for lifetime egg production and egg production rate was only 1.9 mg day^−1^ and the angle (θ) between their position vectors (2.02 deg) was not significantly different from 0 deg (Table 3). We found no significant differences in the linear and quadratic effects of protein and carbohydrate intake between lifetime egg production and egg production rate (Table 3). The correlational effect of protein and carbohydrate intake differed significantly between these two response variables, but this difference was not strong enough to produce a significant overall difference in the effects of protein and carbohydrate intake between them (Table 3). The 95% CR of the peaks for lifetime egg production and egg production rate formed triangular areas in the nutrient space (Fig. 2C,D) and overlapped substantially within the range of protein intake from 5.39 to 15.73 mg day^−1^ and carbohydrate intake from 0.99 to 8.32 mg day^−1^.

**Table 3.** Pairwise comparisons of nutritional landscapes mapped for female lifespan, lifetime egg production, egg production rate and reproductive lifespan in *T. molitor* beetles

Trait pairs	*F*				*d* (mg day^−1^)		θ (deg)	
	Linear	Quadratic	Correlational	Overall	Estimate	95% CI	Estimate	95% CI
Lifespan versus:								
lifetime egg production	15.7***	0.23^n.s^	16.24***	10.62***	3.26	3.15, 3.33	5.98	−14.31, 26.28
egg production rate	19.83***	1.26^n.s^	3.39^n.s.^	9.95***	5.02	4.82, 5.27	7.75	−10.54, 26.04
reproductive lifespan	11.77***	2.11^n.s^	5.81*	7.41***	1.59	1.04, 2.38	2.41	−16.52, 21.34
Lifetime egg production versus:								
egg production rate	1.37^n.s.^	1.44^n.s.^	4.24*	2.06^n.s.^	1.90	1.73, 2.13	2.02	−20.37, 24.43
reproductive lifespan	0.30^n.s.^	1.06^n.s.^	2.59^n.s.^	1.09^n.s.^	2.10	1.75, 2.47	3.81	−19.19, 26.82
Egg production rate versus:								
reproductive lifespan	2.36^n.s.^	1.88^n.s.^	0.25^n.s.^	1.86^n.s.^	4.70	3.58, 6.17	5.30	−16.28, 26.89

*F*-ratios from partial *F*-tests comparing the linear, quadratic and correlational effects of protein and carbohydrate intake on lifespan, lifetime egg production, egg production rate and reproductive lifespan in female *T. molitor* beetles are presented, along with the Euclidean distance (*d*, in mg day^−1^) between the nutritional optima for two traits under comparison and the angle (θ, in degrees) between their position vectors. 95% CI, 95% confidence interval (lower, upper); n.s. not significant; *P<0.05, ***P<0.001.

Pairwise comparison between egg production rate and female lifespan revealed significant differences in the linear effects of protein and carbohydrate intake, leading to significant overall differences in the effects of protein and carbohydrate intake on these response variables (Table 3). Accordingly, the nutritional landscapes mapped for egg production rate and female lifespan differed in shape (Fig. 1B,D). The distance (*d*) between the peaks was 5.02 mg day^−1^ and the angle (θ) between their position vectors was 7.75 deg. Despite this divergence, the directions of the two position vectors were not significantly different, as the 95% CI of the angle included 0 deg.

When comparing the effects of protein and carbohydrate intake between lifetime egg production and female lifespan (Fig. 1B,C), we detected significant differences in the linear and correlational effects of these macronutrients, resulting in significant overall differences in the nutritional effects between the two response variables (Table 3). However, the distance between the peaks for lifetime egg production and female lifespan was closer (3.26 mg day^−1^) than that between those for egg production rate and female lifespan (see above). Similarly, the angle (θ) between the position vectors for lifetime egg production and female lifespan was also smaller (5.98 deg) than that observed between those for egg production rate and female lifespan (see above).

### Reproductive lifespan

In females, the length of reproductive period or reproductive lifespan (mean±s.e.m.: 69.3±5.14 days) was ca. 31.2 days shorter than their actual lifespan (100.5±4.40 days) across the 35 diet treatments. Reproductive lifespan was significantly affected by the intake of both protein and carbohydrate (Fig. 1E). As indicated by significant positive linear and negative quadratic terms for protein and carbohydrate intake (Table 1), reproductive lifespan increased with increasing protein and carbohydrate intake and exhibited a peak in the nutritional landscape (Fig. 1E). This peak occurred at a P:C ratio of 1:1.06 (protein 7.77 mg day^−1^, carbohydrate 8.28 mg day^−1^) and at high P+C intake. As with actual lifespan, reproductive lifespan decreased as the P:C ratio either increased or decreased from this optimal P:C ratio and also as the P+C intake decreased (Fig. 1E).

When comparing actual and reproductive lifespan in females, we detected significant differences in the linear and correlational effects of protein and carbohydrate intake between these two response variables (Table 3), reflecting that reproductive lifespan dropped more rapidly than actual lifespan as the P:C ratio decreased below its optimum (Fig. 1B,E). Although the optimal P:C ratio was slightly higher for reproductive lifespan than for actual lifespan, the angle (θ) between their position vectors was small (2.41 deg) and not significantly different from 0 deg (Fig. 1E, Table 3). The 95% CR of the peak for reproductive lifespan formed a broad quadrangular region in the nutrient space, spanning protein intake above 5.03 mg day^−1^ and carbohydrate intake below 10.00 mg day^−1^ (Fig. 2E), and overlapped with that of the peak for actual lifespan (Fig. 2B).

Pairwise comparisons between reproductive lifespan and either of the two measures of reproductive performance (i.e. lifetime egg production and egg production rate) revealed no significant differences in the linear and non-linear effects of protein and carbohydrate intake across these response variables (Table 3), suggesting that their nutritional landscapes were largely similar in shape (Fig. 1C–E). Consistent with this result, the 95% CR of the peak for these three traits broadly overlapped in the nutrient space, particularly in regions characterized by high protein and low carbohydrate intake (Fig. 2C–E).

**Fig. 3.**
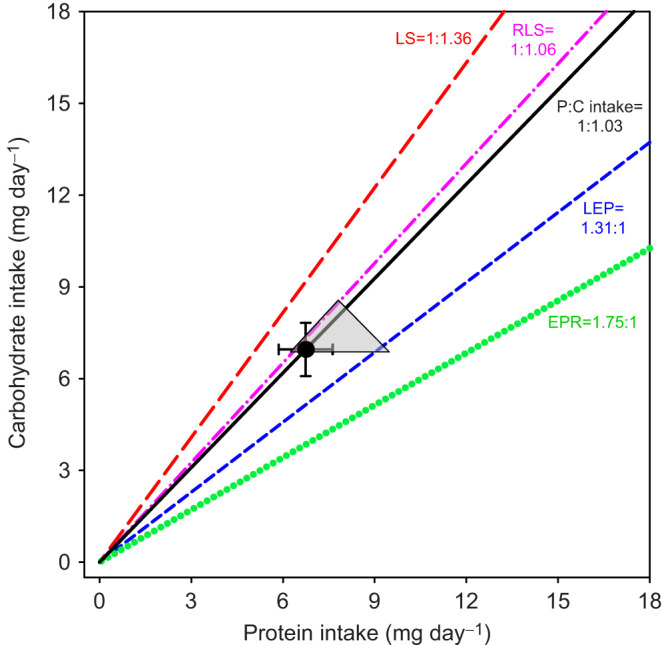
**Bivariate means (±s.e.m.) of self-selected protein and carbohydrate intake by female *T. molitor* beetles in a food choice assay.** The grey triangular area represents the region in the nutrient space where the 95% confidence regions of the peaks for female lifespan (LS), lifetime egg production (LEP), egg production rate (EPR) and reproductive lifespan (RLS) overlap. The black solid line indicates the self-selected protein:carbohydrate (P:C) intake ratio. Coloured lines represent the P:C ratios that maximize specific life-history traits (red long-dashed line, LS; blue short-dashed line, LEP; green dotted line, EPR; pink dash-dotted line, RLS).

Collectively, our inspection of the 95% CR of the peaks for actual lifespan, lifetime egg production, daily egg production rate and reproductive lifespan in females revealed that all four traits overlapped – albeit narrowly – around the centre of the nutrient space (Fig. 3). The region where all four CRs converged formed a small triangular area, with vertices at (protein 7.99 mg day^−1^, carbohydrate 8.35 mg day^−1^), (6.48 mg day^−1^, 6.85 mg day^−1^) and (9.41 mg day^−1^, 6.86 mg day^−1^).

### Macronutrient self-selection

There were no significant sex-specific differences in the amount of protein and carbohydrate selected by *T. molitor* beetles during the feeding period (ANOVA, protein: *F*_1,46_=0.33, *P*=0.571; carbohydrate: *F*_1,46_=0.42, *P*=0.519; MANOVA: Pillai's trace=0.0091, *F*_2,45_=0.21, *P*=0.813). The average self-selected P:C ratio was 1:1.02 (protein 6.41 mg day^−1^, carbohydrate 6.55 mg day^−1^) in males and 1:1.03 (protein 6.74 mg day^−1^, carbohydrate 6.96 mg day^−1^) in females (Fig. 3). These self-selected ratios did not significantly differ from a 1:1 ratio (one-sample *t*-test, male: *t*_23_=0.38, *P*=0.709; female: *t*_23_=0.40, *P*=0.696).

It is important to note that the intake point representing the self-selected amounts of protein and carbohydrate by females over the food choice assay was located inside the triangular region where the peaks of all four life-history traits overlapped (Fig. 3), and was positioned very close to its left vertex. The nutrient rail corresponding to the self-selected P:C ratio also traversed this triangular region. Among the optimal P:C ratios identified for four life-history traits, the self-selected P:C ratio most closely matched that of reproductive lifespan (Fig. 3).

## DISCUSSION

Here, we present the first and most comprehensive analysis of the effect of protein and carbohydrate intake on lifespan, reproduction and their trade-off in a beetle – an insect group that has been underrepresented in studies of nutritional interventions in lifespan and ageing in insects. This study also provides valuable information on the lifespan of the *T. molitor* beetle. We show that *T. molitor* beetles can live as long as ca. 183–197 days depending upon the ingested ratio of protein and carbohydrate – a lifespan much longer than previously reported for this species (Armitage et al., 2003; Krams et al., 2016; McConnell and Judge, 2018; Ribeiro et al., 2018).

The nutritional landscapes mapped for lifespan clearly demonstrated that the lifespan of *T. molitor* beetles increased with increasing caloric intake, contradicting the long-standing view that the restriction of calorie intake is responsible for extending lifespan (Masoro, 2005; Mair and Dillin, 2008; Speakman and Mitchell, 2011). More importantly, we found that lifespan was profoundly influenced by the balance of protein and carbohydrate intake in *T. molitor* beetles. This corroborates the prevailing notion that the balance between these macronutrients is a key determinant of lifespan and ageing in insects (Lee et al., 2008; Piper et al., 2011; Nakagawa et al., 2012; Le Couteur et al., 2016; Simpson et al., 2017; Moatt et al., 2019). When protein was ingested in excess relative to carbohydrate, a marked reduction in lifespan was observed in both male and female beetles, as has been demonstrated for a number of insects. Although its exact underlying mechanism remains elusive, the lifespan-shortening effect of high protein intake could be driven by elevated production of toxic nitrogenous waste, increased generation of mitochondrial reactive oxygen species, changed immune function and/or altered nutrient-sensing pathways, such as insulin/insulin-like growth factor and target of rapamycin signalling pathways (Kapahi et al., 2004; Sanz et al., 2004; Tatar et al., 2014; Mirzaei et al., 2014).

Contrary to the general pattern of longer female longevity observed in insects (Fox et al., 2003; Bonduriansky et al., 2008; Sielezniew et al., 2020), males lived longer than females in *T. molitor* beetles. Despite this sex-specific difference in overall lifespan, the lifespan of male and female *T. molitor* beetles did not respond differently to protein and carbohydrate intake, with their maximal lifespan occurring at nearly identical P:C ratios (1:1.38 for males and 1:1.36 for females). These results imply that the sensitivity of nutrient-sensing pathways influencing lifespan may not differ between sexes in this species. A recent phylogenetic comparative analysis by Morimoto (2024) found marked sex-specific differences in optimal P:C ratios for lifespan in Orthopterans – particularly crickets−where females require greater carbohydrate intake than males to maximize lifespan (Maklakov et al., 2008; Harrison et al., 2014; Rapkin et al., 2017; Hawkes et al., 2022). However, such differences were not evident in Dipterans (Jensen et al., 2015; Malod et al., 2017; Morimoto, 2024). Further studies across a broader range of beetle species are required to assess whether the lack of sex-specific differences in optimal P:C ratios for lifespan is characteristic of Coleopterans.

As has been observed in a number of insects (Lee et al., 2008; Maklakov et al., 2008; Simpson and Raubenheimer, 2012; Jensen et al., 2015), we found that a moderate increase in protein intake enhanced both daily and lifetime female reproductive performance in *T. molitor*. This positive effect of moderately high protein intake on reproduction can be attributed to the fact that protein is the main raw material for egg production and also stimulates vitellogenesis and oogenesis in insects (Wheeler, 1996; Mirth et al., 2019). However, when protein intake exceeded the optimal level, egg production declined, indicating the potential toxicity of excessive protein consumption (Lee et al., 2008).

Numerous studies, mostly from flies and crickets, have consistently documented a substantial divergence between the nutritional optima for lifespan and female reproductive traits, with lifespan being maximized at much lower or more carbohydrate-biased P:C ratios than those that maximized female fecundity (Lee et al., 2008; Maklakov et al., 2008; Fanson et al., 2009; Harrison et al., 2014; Archer et al., 2015; Jensen et al., 2015; Malod et al., 2017; Rapkin et al., 2017; Semaniuk et al., 2018; Jang and Lee, 2018; Ng et al., 2018, 2019; Carey et al., 2022; Hawkes et al., 2022). Identifying the occurrence and strength of this divergence has been a central focus of recent research on ageing and life-history evolution, because it enables a quantitative assessment of whether, and to what extent, the intake of multiple macronutrients mediates the trade-off between these two key fitness components (Morimoto and Lihoreau, 2019; Morimoto et al., 2023). Using 35 diets encompassing a broad range of protein and carbohydrate content, we were able to detect a divergence between the nutritional optima for female lifespan and the rate of egg production in *T. molitor* beetles – an outcome that was not observable in an early study using only three isocaloric diets (Rho and Lee, 2016). These results indicate the presence of a nutritional conflict between lifespan and reproduction in achieving their respective trait maxima in *T. molitor*.

When we compared our results with those from previous studies on crickets and flies, we noticed some interspecific differences in the magnitude of nutritional divergence between lifespan and egg production rate. In decorated crickets (*Teleogryllus commodus*), lifespan was maximized at an extremely low P:C ratio of 1:8 whereas egg production rate was maximized at a P:C ratio of 1:1 (Rapkin et al., 2017). Similarly, in *Drosophila melanogaster*, it was recently reported that the P:C ratios that maximized lifespan and egg production rate were 1:15.88 and 1:1.22, respectively (Carey et al., 2022). Compared with these two species, the divergence between the nutritional optima for female lifespan and egg production rate was less pronounced in *T. molitor* beetles (1:1.36 for lifespan and 1.75:1 for egg production rate), which was also evidenced by their overlapping peak regions and position vectors. These results indicate a weaker nutrient-mediated trade-off between lifespan and reproduction in *T. molitor* beetles compared with crickets and *Drosophila* flies.

What could explain the variation in the strength of this trade-off among these insect species? While the differences in the P:C ratios that maximized daily egg production were relatively small among *T. molitor* beetles (1.75:1), crickets (1:1; Rapkin et al., 2017) and *Drosophila* flies (1:2; Carey et al., 2022), the P:C ratios that maximized lifespan varied considerably among them. For example, lifespan was maximized at a much higher P:C ratio in *T. molitor* beetles (1:1.36) compared with crickets (1:8) and *Drosophila* flies (1:15.88). These results suggest that species-specific differences in the optimal P:C ratio for lifespan are the primary driver of this observed variation in the strength of the nutrient-mediated lifespan–reproduction trade-off across these species. Why *T. molitor* beetles maximize their lifespan at a relatively higher P:C ratio than other insects remains an open question. Further research is needed to determine whether this pattern reflects fundamental differences in the sensitivity of the nutrient-sensing pathways regulating lifespan and ageing among these three species.

Because of their high optimal P:C ratio for lifespan, *T. molitor* beetles – unlike crickets and *Drosophila* flies – experienced shortened lifespan when consuming extremely carbohydrate-biased or low P:C diets. This negative effect of extremely low P:C ratio on *T. molitor* lifespan may result from obesity-related metabolic dysfunction caused by excessive carbohydrate intake (Skorupa et al., 2008; Abrat et al., 2018), possibly compounded by a failure to meet protein requirements for somatic maintenance and repair. While many insects have been shown to actively eliminate carbohydrate excesses from the body as CO_2_ through elevated respiration rates (Zanotto et al., 1993, 1997), the adults of *T. molitor* have been shown to possess a limited capacity to counteract carbohydrate surpluses via this post-ingestive regulatory mechanism (Urrejola et al., 2011). This may explain why *T. molitor* beetles experienced a shortened lifespan, possibly due to obesity-related health risks, when consuming carbohydrate-biased diets.

Similar to actual lifespan, the duration of the reproductively active period (i.e. reproductive lifespan) in *T. molitor* beetles was highly nutrient dependent, forming a convex nutritional landscape with a distinct peak at a P:C ratio near 1:1 (Maklakov et al., 2009; Jensen et al., 2015). However, actual and reproductive lifespan responded somewhat differently to protein intake. For instance, when protein intake became extremely limited (below 3 mg day^−1^), reproductive lifespan declined more rapidly than actual lifespan. Because of this difference, *T. molitor* beetles consuming extremely carbohydrate-biased diets ceased their egg laying early in life and spent nearly 60% of their remaining lifespan in a non-reproductive state. It remains unclear whether egg production in protein-limited *T. molitor* beetles ceases permanently or can resume upon the reintroduction of protein-rich diets. Whatever the case, this early termination of egg production induced by low protein intake may be mediated by reduced levels of juvenile hormone in the haemolymph, a key regulator of vitellogensis and oogenesis in insects (Riddiford, 2012; Klowden, 2013).

As females require more protein for egg production than males in insects, females are generally expected to prefer a diet with a higher P:C ratio than males when offered a food choice (Lee et al., 2013; Camus et al., 2018; but see Maklakov et al., 2008; Rapkin et al., 2017). Contrary to this prediction, both male and female *T. molitor* beetles self-selected a P:C ratio close to 1:1 (male: 1:1.02; female: 1:1.03). For females, this self-selected P:C ratio simultaneously supported maximal lifespan and all measured reproductive traits. These findings add to the growing body of research supporting the adaptive hypothesis that insects can maximize organismal fitness by optimizing the intake of multiple macronutrients (Lee et al., 2008; Jensen et al., 2012; Raubenheimer and Simpson, 2018). Interestingly, the self-selected P:C ratio closely matched the ratio that maximized female reproductive lifespan but diverged from the one that maximized the rate of egg production. This pattern of nutrient selection suggests that female beetles prefer a diet that prioritizes extending the duration of their reproductive period over maximizing daily egg output. Such a strategy – laying eggs over a longer period but at a slower rate – may be adaptive, as rapid egg production is energetically and nutritionally costly and may therefore compromise egg quality.

In summary, lifespan and female reproductive traits were profoundly influenced by dietary P:C balance in *T. molitor* beetles. These fitness components were maximized at different P:C ratios, indicating the occurrence of a nutrient-mediated trade-off between them. However, the magnitude of this trade-off appeared to be weaker in *T. molitor* than in other insect species studied to date. Further research across diverse beetle species is needed to determine whether this weak or moderate nutritional conflict between lifespan and reproduction is a general feature of Coleopterans. Finally, we hope that our study will pave new avenues for establishing *T. molitor* as a promising model organism in lifespan and ageing research.
